# Agency in avoidant personality disorder: a narrative review

**DOI:** 10.3389/fpsyg.2023.1248617

**Published:** 2023-09-18

**Authors:** Andrea Varga Weme, Kristine Dahl Sørensen, Per-Einar Binder

**Affiliations:** ^1^Group Therapy Team, Voss Outpatient District Psychiatric Unit NKS Bjørkeli AS, Voss, Norway; ^2^Department of Clinical Psychology, Faculty of Psychology, University of Bergen, Bergen, Norway; ^3^Group Therapy Team, Aust-Agder Country Outpatient Psychiatric Unit, Sørlandet Hospital, Arendal, Norway

**Keywords:** avoidant personality disorder, cluster C personality disorders, agency, client factors, narrative review

## Abstract

**Objectives:**

Avoidant personality disorder (AvPD) is a highly prevalent personality disorder, especially in clinical settings, yet scarcely researched. People diagnosed with AvPD have severe impairments in functioning and suffer greatly, yet we still lack meta-analytic evidence for therapy and only a few RCTs are conducted. Patient factors are the most important for outcome in therapy, in general. Lack of agency might be a core deficit in people diagnosed with AvPD. Their conditions might be improved if we understand their agency better. We review previous research regarding psychological mechanisms and interpersonal relationships that facilitate or hinder agency in AvPD in daily life and psychotherapy.

**Methods:**

Summarizing original literature in a narrative review with reflexive thematic analysis.

**Results:**

People diagnosed with AvPD seem to have significant impairments in their sense of agency due to a lack of emotional awareness, an overweight of inhibiting vs. activating emotions, and difficulties regulating emotions. Difficulties also seem related to high levels of attachment avoidance and fear, creating strong ambivalence in social needs, in addition to a strong tendency to subordinate to others. A weak sense of self with a poor narrative, self-doubt, and harsh self-critique makes a reflexive and intentional stand increasingly difficult for these people.

**Conclusion:**

This review gives a clinically meaningful understanding of core strengths and deficits in the personality functioning of AvPD that can help clinicians map out important therapeutic work, identify barriers to client-agency in therapy, and work through relational difficulties in the therapeutic alliance.

## 1. Introduction

How can we understand the role agency plays in avoidant personality disorder (AvPD)? Agency is defined as being a motivated being who has goals, motives, values, and strives toward them. It is a part of what constitutes a self together with social roles and the stories we construct about our lives (McAdams, 2013). Agency also entails a sense of ownership of your own emotions, thoughts and actions and an awareness that they stem from you and not from others. This sense of agency is an important and separate but correlated part of self-reflective functioning together with emotional awareness, the ability to distinguish between fantasy and reality and the ability to integrate a range of different perspectives. We refer here to a higher order conscious process of detecting and describing aspects of the self, that builds on but should be distinguished from agency in neuroscience – which refers to an unconscious sense of your movements belonging to you and that they are in your control (Dimaggio et al., 2009). Agency requires both an action component and a self-reflexive component relating the actions to the self and inner motives (McAdams, 2013). Difficulties with agency seems to play an important role in many psychopathologies, such as difficulties with regulating self-esteem (McAdams, 2020), or difficulties with recognizing thoughts and actions as their own – attributing them to external forces (Dimaggio et al., 2009). When lacking access to their own motives, people can become driven by how they think others perceive them and expect from them. This excessive third-person perspective can be found in people with social anxiety (McAdams, 2020). In this review we map out how people with AvPD might experience their agency.

Psychotherapy is best practice for AvPD (Weinbrecht et al., 2016; Lampe and Malhi, 2018), so what implications might client-agency have for psychotherapy with this patient group? We define agency in psychotherapy from an existential view, as a cycle between unaware intentional actions and moments of self-awareness and reflection informing each other (Rennie, 2000, 2001; Mackrill, 2009). We see clients as cross-contextual agents who seek to fulfill their desires in many ways, where therapy is but one limited context. Reflection is viewed as an aspect of this (Mackrill, 2009). Agency in psychotherapy entails a relationship with the self (also in daily life), a relationship with the therapists, and a relationship with the therapists’ techniques (Rennie, 2000, 2001; Mackrill, 2009; Lavik et al., 2018). What patients do in their daily life and how they contribute in the psychotherapeutic process is the most significant factor for the outcome of psychotherapy in general (Bohart and Tallman, 2010; Lambert, 2013). It is important to acknowledge though, that outcome of psychotherapy is an intricate interplay between both patients, therapists, the relationship, and therapeutic method – that works together in inseparable ways clinically (Norcross and Lambert, 2011). Despite of the strong importance of the patients’ contribution to change, we continue to reduce patients’ role in research to reporting symptom change and rarely investigate how patients themselves contribute to change processes. This might be due to a bias of overvaluing the therapists’ methods in effect studies, also leading therapists to focus on the method (Bohart, 2000). It might be particularly important to be aware of this bias with people diagnosed with AvPD as they report feeling handled by their therapists and that influencing their therapeutic process feels impossible (Sørensen et al., 2019b) – indicating a low sense of agency in therapy. It also seems to be an important barrier for therapists, as they report oscillating between taking over the therapeutic project or being too passive when treating people diagnosed with AvPD (Pettersen et al., 2021) – perhaps struggling to support their patients’ agency.

Given the important role of agency for positive therapeutic outcomes, we perceive this as a potential barrier to successful therapies with AvPD. Hayes and Yasinski (2015) illustrate the importance of agency in therapy in their study of patients diagnosed with cluster C personality disorders (cluster C). Positive patterns of thinking, feeling, acting, bodily experiences, and hope for the future at the beginning of therapy predicted more change throughout the therapeutic process. Positive and negative patterns did not correlate, indicating that creating new positive patterns is something different from breaking bad patterns and important with this patient group. Low identity integration is one of the most pronounced personality dysfunctions (Eikenæs et al., 2013) and non-assertiveness is the greatest interpersonal difficulty (Frandsen et al., 2020) in this group, pointing to a lack of agency as a core deficit that contributes greatly to people diagnosed with AvPD’s life struggles in general (Sørensen et al., 2019a). Difficulties with agency could contribute to the psychological pain people diagnosed with AvPD endure. Understanding more about how people diagnosed with AvPD experience agency in daily life and therapy could therefore help therapists understand how to facilitate agency with this group in psychotherapy and greatly improve their quality of life. Metacognitive interpersonal therapy (MIT) has increasing agency as one of their core components, and difficulties with agency is at the center of their understanding of AvPD (Dimaggio et al., 2020; Centonze et al., 2021).

AvPD is a mental disorder under personality disorders (PDs) in the American DSM-5 and World Health Organization ICD-10 diagnostic manuals and defined by at least four of the seven diagnostic criteria: (1) Avoidance of occupational activities that involve interpersonal contact in fear of criticism, (2) unwillingness to get involved with others unless certain of being liked, (3) restraint in intimate relationships in fear of ridicule, (4) preoccupation with being criticized in social situations, (5) inhibition in new interpersonal situations because of feelings of inadequacy, (6) self-view as inferior to others and (7) reluctance toward new activities due to fear of embarrassment. The pattern must be pervasive and culturally deviant, beginning in early adulthood and resulting in functional impairment in several areas, such as work and relationships (World Health Organization, 2004; American Psychiatric Association, 2013).

In the revision of World Health Organization’s diagnostical system, personality disorders (PDs) are no longer diagnosed by category, except from an option to specify borderline personality traits. PDs are instead diagnosed on a continuum of severity of dysfunction in self-functioning and interpersonal functioning, along with possible trait specifiers (World Health Organization, 2023). The same is true for the new understanding of the alternative model in the American diagnostic manual (AMPD) (American Psychiatric Association, 2013). AvPD is no longer a diagnostic category in ICD-11. It is therefore important for researchers and clinicians to recognize the typical patterns of this patient group as self- and relational dysfunction. Agency is an important self-function that is embedded within the ICD-11 and AMPD through the parts of self-function sense of identity and goal directedness (Bach and Eikenæs, 2021; Lind, 2021; Bach et al., 2022). We therefore argue that this summary is relevant to understand and treat these personality problems also after AvPD has ceased to exist as a distinct diagnosis (Bach and Eikenæs, 2021). In ICD-11 avoidant personality problems can be recognized as marked difficulties in self-esteem, intense fear of criticism and rejection, and a compromised ability to work toward goals due to lack of self-confidence when it comes to self-function. Relational functioning is characterized by avoidance of situations that are perceived as too difficult, social isolation, perceiving others as overly critical and turning down opportunities in fear of failure (Bach and Eikenæs, 2021; Bach et al., 2022).

It is a common disorder with a prevalence between 1.2 and 9.3 percent in Western societies (Quirk et al., 2016; Winsper et al., 2020). The clinical prevalence in Norway is about 40 percent (Kvarstein and Karterud, 2013; Kvarstein et al., 2017). AvPD is correlated with chronic depression (Klein et al., 2015), somatic illness, use of health services and social welfare, less education, unemployment, less likelihood of relationships, and loneliness (Jackson and Burgess, 2004; Olssøn and Dahl, 2012). The disorder has a considerable burden of disease (Wilberg et al., 2009) and AvPD can affect treatment outcomes negatively (Kvarstein and Karterud, 2013).

Despite the high prevalence and disease burden, AvPD has inspired little research, although public attention to AvPD has recently increased. Cluster C (the anxious PDs) comprises AvPD, dependent and obsessive-compulsive PDs, although there is increasing evidence that the latter is distinct from the two other disorders and loads on different factors (de Reus and Emmelkamp, 2012). We include studies on cluster C in this review because of the sparse research on AvPD, and the majority of cluster C study populations have AvPD. Some case studies (e.g., Gilbert and Gordon, 2013; Gordon-King et al., 2019) and within-subjects experiments (e.g., Normann-Eide et al., 2015; Boettcher et al., 2019) investigate various therapeutic manuals and settings with promising results. There are some process studies (e.g., Ulvenes et al., 2014; Lilliengren et al., 2019), and only three qualitative studies (Cummings et al., 2011; Sørensen et al., 2019a; Pettersen et al., 2021), and most research on characteristics of AvPD (e.g., Eikenæs et al., 2016; Simonsen et al., 2020). There are only seven randomized controlled trials (RCT) on AvPD or cluster C. All find significant treatment effects (Alden, 1989; Stravynski et al., 1994; Svartberg et al., 2004; Emmelkamp et al., 2006; Borge et al., 2010; Bamelis et al., 2014; de Jong et al., 2018). Emmelkamp et al. (2006) find cognitive behavioral therapy (CBT) significantly more helpful than brief psychodynamic therapy (DT) or waitlist control, while Svartberg et al. (2004) find both approaches equal. Borge et al. (2010) find a reduction in AvPD with both CT and DT residential approaches aimed at social phobia. Alden (1989) and Stravynski et al. (1994) both find CBT in the form of short-term structured social skills training (SST) and exposure therapy (ET) effective. Alden (1989) finds ET effective, with no significant improvement by adding SST or intimacy focus. Stravynski et al. (1994) find SST helpful. Adding *in vivo* training does not add to the effect, but has significantly more dropout. Bamelis et al. (2014) find both schema therapy (ST), therapy as usual, and clarification-oriented therapy to be effective with cluster C. Observer-rated measures were significant, but not self-report measures. ST had significantly better outcomes than the two other treatments. de Jong et al. (2018) report on an RCT studying the effect of outcome feedback with various PDs. Cluster C had better outcomes when the therapists got feedback than when they did not. The same was not true for other PDs.

There are several specific treatment approaches developed for AvPD, cluster C or emotionally inhibited PDs that show promising results (e.g., Arntz, 2012; Gordon-King et al., 2018; Fassbinder and Arntz, 2019; Simonsen et al., 2022), even though we do not have metanalytical evidence for best practice yet (Weinbrecht et al., 2016; Lampe and Malhi, 2018). We wish to summarize the small but diverse research body in a clinically meaningful way, which can inform clinicians and guide future research on how to facilitate agency in this group independent of theoretical orientation. We use cyclical psychodynamics as a theoretical framework to integrate findings and discuss implications for understanding agency in AvPD based on the current research. Cyclical psychodynamics integrate the internal object relation theories of DT and the here- and now learning new social skills of CBT and SST: People’s first relationships create vicious or virtuous circles that can sustain or change personality through new relational transactions in daily life and therapy (Wachtel, 2014). We will use this as a framework to unite languages of different methodological approaches in the discussion and point out where in the cycle they intervene. In this review, we investigate what previous research tells us about what psychological processes and relationships facilitate agency in people diagnosed with AvPD. What type of characteristics or relational dynamics become barriers? What are the implications for psychotherapy?

## 2. Methods

This review followed the narrative review methodology described by Ferrari (2015) as this allows for analyzing overarching themes in published research with different methodological approaches (Snyder, 2019). The study protocol was registered in Prospero (registration number CRD42022340952) (Weme et al., 2022).

### 2.1. Search strategy

The main electronic searches were conducted in July 2022 using the databases PsycINFO MEDLINE and Embase (all at OVID). An additional search was conducted in CINAHL (at EBSCO) in August 2022. Searches in Scandinavian databases and journals such as SveMed+, bibliotek.dk, LIBRIS, Idunn, Nordic Journal of Psychiatry, Journal of the Norwegian Psychological Association, and Matrix were conducted without finding eligible articles. Monthly alerts were set up to ensure that the included literature remained current. One article was added. The main search words were avoidant personality disorder, cluster C personality disorder/patient, and abbreviations AvPD/cluster C. We tried to combine the search words with part two of the search using words such as agency, self-esteem, or sense of self, but this did not comprise all relevant articles, so we left out part two of the search. All authors hand-searched references in the full-text articles, identifying three additional articles.

### 2.2. Search results

The searches identified 4,164 references that were imported to Endnote. After automatic and manual duplication checks, 2,546 unique references were screened.

### 2.3. Data evaluation process

**Figure 1:**
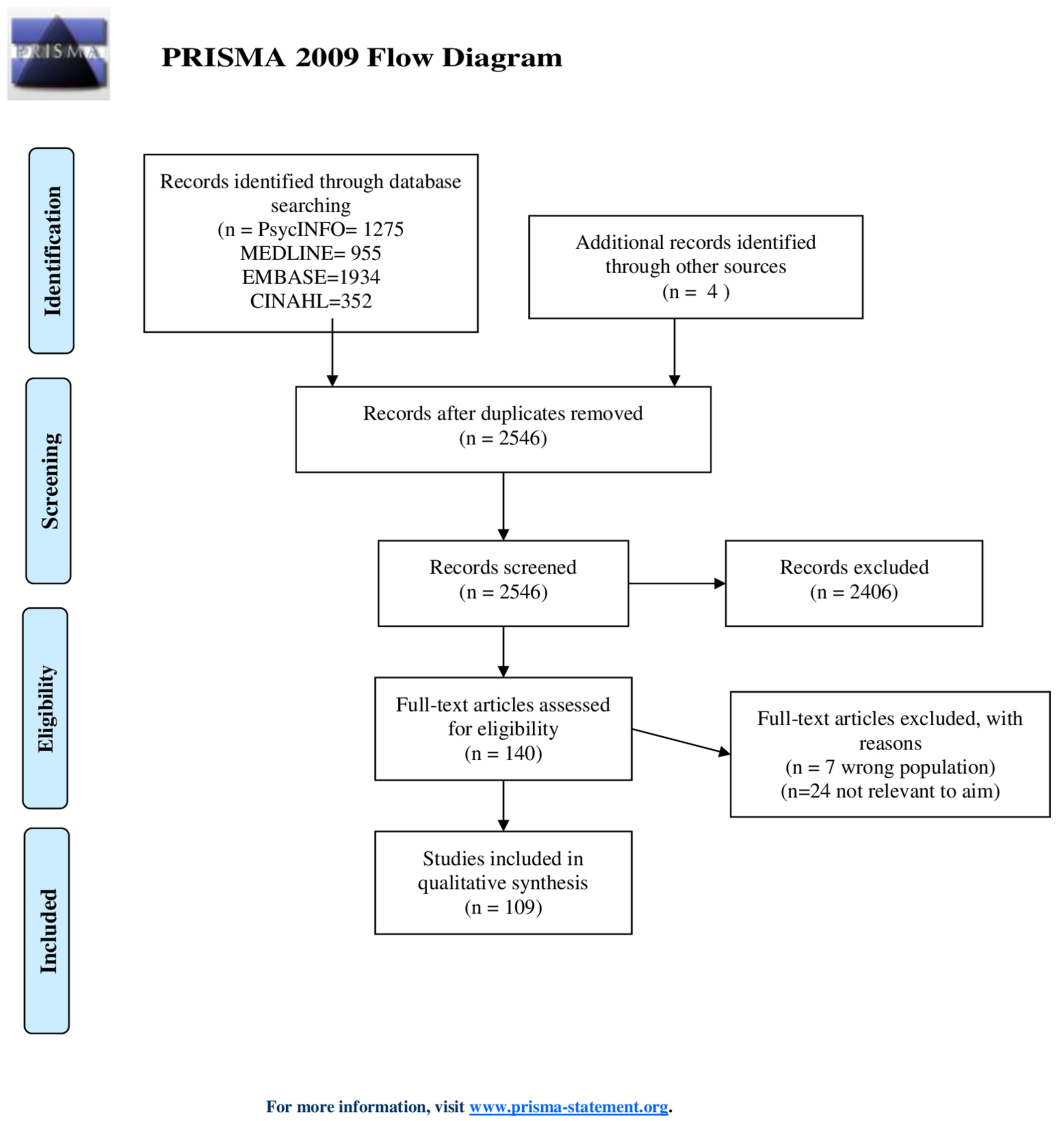
Flowchart of the literature selection process (Moher et al., 2009).

The first author screened titles and abstracts according to the aim of the review and the inclusion and exclusion criteria (Table 1). We used Rayyan as a tool for reference management in the screening process. The third author quality assured the selection by screening 10% of the titles and abstracts blinded for the first author’s decisions. The first author reviewed 70% of the included studies for methodological quality with Critical Appraisal Skills Programme (2022), National Institute for Health Care and Excellence (2012), and Helsebiblioteket (2016)‘s checklists. All authors assessed 140 full-text articles for eligibility by discussing suitability based on the robustness of the research and the degree to which it addressed the research question. All authors discussed disagreements in inclusion until we reached a consensus. The analysis included 109 articles. There was no disagreement in full-text inclusion. Figure 1 describes the selection process using a PRISMA flow chart.

**Table 1:** Inclusion and exclusion criteria.

Inclusion	Exclusion
Scientific original literature such as peer-reviewed articles and dissertations	Case studies without measurements, comments, non-peer-reviewed books and reviews
People with avoidant personality disorder/problems	
Youths and adults (13 years or above)	Younger than 13 years of age
Agency in a population diagnosed with AvPD in daily life or in psychotherapy	Population diagnosed with AvPD, but no information about agency or interpersonal functioning in daily life or psychotherapy
Other functions related to agency in a population diagnosed with AvPD such as affect consciousness, sense of self, self-efficacy, activating and inhibiting affects, goal directedness, avoidance, inhibition, subordination or procrastination	
Interpersonal function in a population with AvPD such as relationships, psychosocial function, metacognition/mentalization, empathy and intimacy in daily life or psychotherapy	
Agency or interpersonal functioning in a wider population applicable to people diagnosed with AvPD such as cluster C PD	Agency or interpersonal functioning in a population of other specific personality disorders or in PDs in general not specifically related to AvPD
Scandinavian, German or English language	A language we cannot read

### 2.4. Analysis

The authors have a hermeneutic phenomenological stance (Binder et al., 2012), trying to understand how people diagnosed with AvPD might experience their agency, and how this might affect their therapeutic process. We also wish to be transparent and reflective about how our backgrounds influence the interpretations in this review. All authors are both clinical psychologists and researchers. We have theoretical backgrounds in DT, mentalization-based therapy (MBT), ST, family therapy, emotion-focused therapy, and mindfulness-based interventions. All authors have an integrative stance on therapeutic methods. The first author and the second author work in specialized group-therapy teams with AvPD. We analyzed the articles with reflexive thematic analysis (Braun and Clarke, 2019; Finlay, 2021) combining findings from the different articles to construct themes to inform the research questions. First, the first author read 70% of the eligible full-text articles iteratively, tentatively constructing initial codes with an inductive approach based on the pattern of findings from the different articles, relating to the objectives of psychological processes or relationships that could facilitate or be barriers to agency. A second round of analysis was undertaken where all authors discussed the preliminary codes and went back and forth between reading the full-text articles, regrouping, and synthesizing coherent themes. This process proceeded until we agreed upon final themes. We connected the themes with the overarching theoretical context of cyclical psychodynamics in a third round of analysis. The process was not linear, and the researchers went back and forth between the three stages of analysis. The first author kept a reflective journal of emerging thoughts and ideas throughout the process to strengthen the analytic process (Green et al., 2006). To strengthen the researchers’ reflexivity two co-researchers with first-hand experience of AvPD and therapy and two clinicians working with AvPD read the final themes and gave feedback in the last round of analysis. When researchers with different backgrounds discuss findings, it is easier to become aware of preconceptions, which may open alternative ways of interpreting the data (Veseth et al., 2017).

## 3. Results

**Figure 2:**
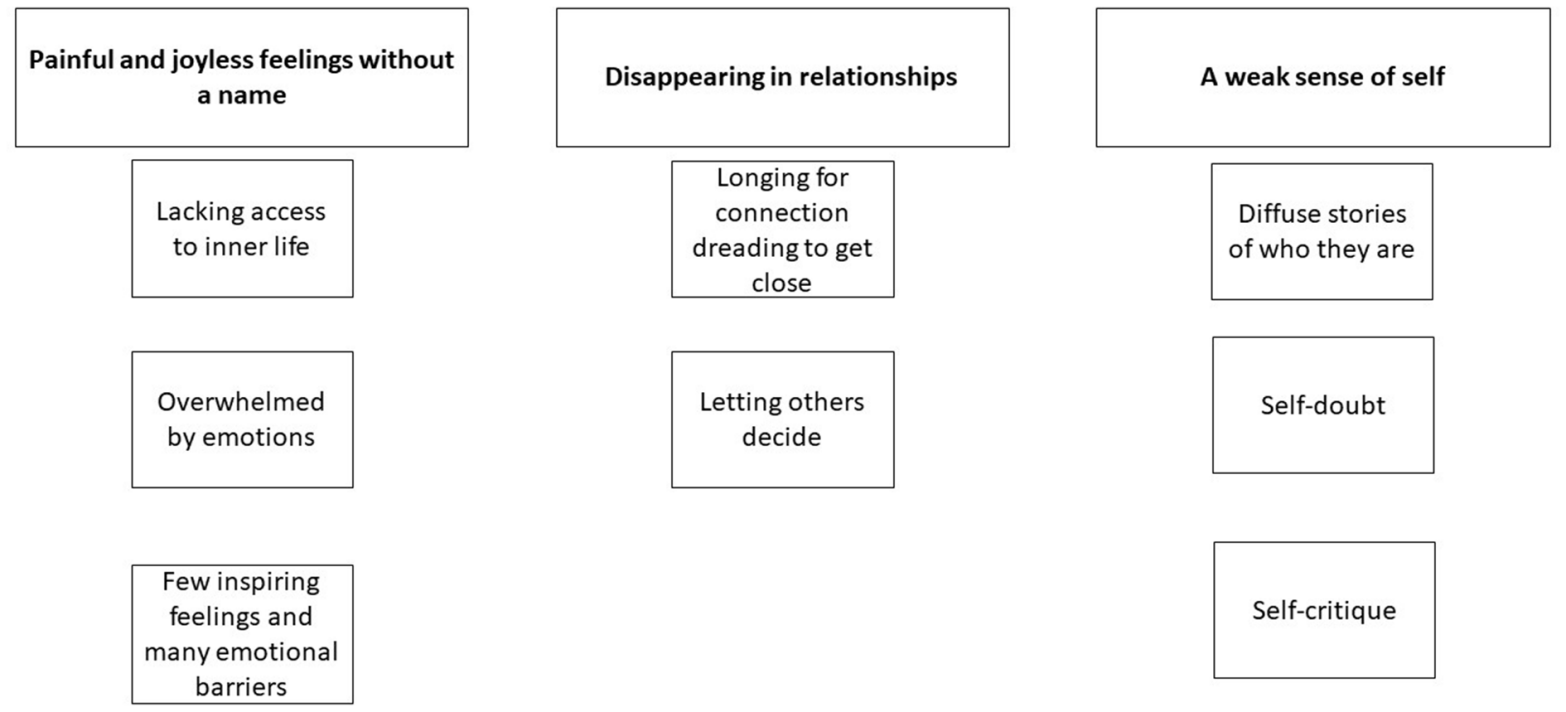
Overview of main themes and subthemes.

Below we describe three main themes: How people diagnosed with AvPD typically experience their emotions, relationships, and themselves – with related subthemes. First, we describe how every subtheme transpires in daily life. At the end of each subtheme, we discuss how this appears in therapy and possible interventions. An overview of the main themes and subthemes is presented in Figure 2.

### 3.1. Painful and joyless feelings without a name

This theme is about how people diagnosed with AvPD experience emotions – as these are important driving forces behind agency and sources of self-knowledge (Normann-Eide, 2020). We argue here that people diagnosed with AvPD have fundamental difficulties in this area, through the subthemes lack of access to inner experiences, low tolerance for emotions, and few positive and many negative emotions.

#### 3.1.1. Lacking access to inner life

Difficulties with identifying and expressing internal experiences such as emotions are found in many PDs (e.g., Johansen et al., 2013; Frederiksen et al., 2021). This is referred to with different but similar concepts like lack of affect consciousness (Johansen et al., 2013), alexithymia (Nicolo et al., 2011; Coolidge et al., 2013; Simonsen et al., 2020), or difficulties with monitoring (Moroni et al., 2016). We choose to use the word alexithymia for these deficits. Alexithymia seems to be a particularly important deficit for understanding AvPD (Nicolo et al., 2011; Coolidge et al., 2013; Johansen et al., 2013; Moroni et al., 2016; Simonsen et al., 2020; Frederiksen et al., 2021). Specifically, they have greater difficulties with conceptual expression – forming a mental concept of their emotions and verbalizing them (Johansen et al., 2013). The extent of these difficulties varies, and they are correlated with poorer personality functioning (Simonsen et al., 2020). Alexithymia could be a specific characteristic of AvPD. Depressed patients who are also diagnosed with cluster C show more stability in this deficit than depressed patients without a diagnosis of cluster C (Honkalampi et al., 2001) and it correlates with difficulties in mentalizing – the ability to verbalize inner states, while the same is not true for people with BPD (Johansen et al., 2018). Alexithymia is related to cluster C when a person has difficulties with mindreading, but not necessarily in people without mindreading difficulties (Lysaker et al., 2014). Somatic symptoms (Olssøn and Dahl, 2012) are more prevalent in this group and can be without physiological explanations (Caseras et al., 2001; Simonsen et al., 2019). This may be due to difficulties with inferring bodily sensations as emotions (Gordon-King et al., 2019). Some perceive expressing emotion as a weakness (Hyman and Schneider, 2004; Jovev and Jackson, 2004; Gordon-King et al., 2019) and show a tendency to avoid and inhibit emotions (Currie et al., 2017; Flink et al., 2019; Kunst et al., 2020).

Literature on therapy also describes difficulties with experiencing and expressing emotion: Describing patients as intellectualizing, externalizing, overusing metaphors (Gordon-King et al., 2017, 2019) and overthinking as emotional coping strategies (van Rijsbergen et al., 2015). Not being able to verbalize emotions in therapy can give a sense of failure (Gordon-King et al., 2019). Therapists might react with anger or hopelessness in the face of silent answers from their patients (Ogrodniczuk et al., 2011; Simonsen et al., 2019; Pettersen et al., 2021). Therapists can react to avoidance as dismissal and withdraw or take too much action. Being aware of their emotional reactions can be a good compass for therapists to address alliance ruptures and helps to maintain an empathic stance (the same skill therapists try teaching patients) (Cummings et al., 2011). The literature suggests several ways of working to increase awareness and expression of emotion. A therapeutic stance of reflection and empathy to encourage disclosure (Hyman and Schneider, 2004) and to identify and verbalize internal experiences as they occur in therapy (Dimaggio et al., 2017; Simonsen et al., 2019; Bachrach and Arntz, 2021). MBT and MIT involve practicing expressing inner states, and they report changes in alexithymia (Normann-Eide et al., 2015; Gordon-King et al., 2019; Simonsen et al., 2022; Wilberg et al., 2023). Hyman and Schneider (2004) report an increased sense of self in their case study after practicing emotional expression in daily life. Process studies support this as orienting patients toward affect activates more affects, and this correlates with a better sense of self, but the effect is bigger for those with a better sense of self at baseline (Ulvenes et al., 2014). Changes in alexithymia also correlate with self-respect, self-reflection (Simonsen et al., 2022), self-esteem, identity integration, frustration tolerance, feeling joy, and viewing life as stable, integrative, and meaningful (Normann-Eide et al., 2015). Yet, therapists use prescriptive interventions, and interventions exploring the past more, not exploratory experiential interventions with cluster C compared to other pathologies (Kolden et al., 2005).

#### 3.1.2. Overwhelmed by emotions

Higher occurrence of self-harm, experiencing more emotional arousal, affective instability and easily becoming overwhelmed is also typical (Snir et al., 2015, 2017). Overwhelming and painful emotions may be linked to difficulties with experiencing emotions, as they describe diverting themselves when alone, in fear of being engulfed in feelings of longing and fear (Sørensen et al., 2019a), and dissociative experiences are reported in advance of self-harming (Snir et al., 2015). People diagnosed with AvPD who can identify emotions have better frustration tolerance, emotional regulation, and self-constraint (Simonsen et al., 2020).

People diagnosed with AvPD report seeking help when their avoidant strategies no longer give them sufficient relief (Sørensen et al., 2019b). Practicing tolerating emotions is suggested to be an important therapeutic factor (Gordon-King et al., 2017). Acceptance and commitment therapy (ACT) combined with dialectical behavioral therapy (DBT) describe avoidance of painful emotions as an important hinder to agency. They intervene with psychoeducation, mindfulness, acceptance and diffusion cognitive techniques, relaxation, and breathing exercises to develop a tolerance for painful emotions, reporting more emotional stability in their case study (Chan et al., 2015). AvPD is associated with negative emotions also when controlling for cognitive appraisal style (Jarnecke et al., 2017). This may suggest that it is important to help patients regulate these emotions in more ways than cognitive restructuring. In order not to overwhelm the participants, Bo et al. (2019) structure their group therapy with adolescents with little emotionally demanding topics in the beginning, before group cohesion develops. Practicing regulating emotions with relaxation and desensitization before exposure to avoided social situations could be important if overwhelming emotions stop people from trying new things (Renneberg et al., 1990). On the other hand, therapists might feel too protective of their patients, being careful not to disclose their vulnerabilities (Colli et al., 2014), and experience patients’ emotions (such as guilt) as disproportionate (Pettersen et al., 2021). This could create urges to remove the emotional pain and make it difficult to accept and validate the patients’ emotional experience (Pettersen et al., 2021). This may thwart feeling understood and accepted, and developing a capacity of trusting their emotional reactions. Strauss (2001) found better results from CBT when emotional arousal in sessions was high but in a good alliance.

#### 3.1.3. Few inspiring feelings and many emotional barriers

Less positive emotions (Meyer, 2002; Hummelen et al., 2007) such as interest, excitement (Johansen et al., 2013; Frederiksen et al., 2021), and joy (Eikenæs et al., 2013; Karterud et al., 2016; Simonsen et al., 2019) are found in populations of AvPD. This is also supported by the finding that happy child mode in ST is the mode most strongly and negatively correlated with AvPD. Happy child mode entails being playful and spontaneous (Lobbestael et al., 2008). These emotions motivate exploration of the world, help form and maintain relationships, find out likes and dislikes, test out new behavior, and learn new things. Playfulness and interest are important emotions for agency as they give you a feeling of what you want, and an opportunity for trying it out (play) (Normann-Eide, 2020).They are all positive emotions behind the behavioral activation system (BAS) (Gray, 1987). They give motivation in doing things to increase pleasure and drive toward rewards. We may assume that having little access to BAS can make it difficult to be agents, as this could make wishes less accessible and give less drive and confidence in trying new things. People diagnosed with AvPD also have significantly less access to feelings of contempt (Johansen et al., 2013) and anger (Hyman and Schneider, 2004; Karterud et al., 2016). Other studies do not find this (Frederiksen et al., 2021). Having difficulties with experiencing such driving negative emotions can make agency difficult, as these feelings are important in setting boundaries and in assertiveness.

More maladaptive schemas of over-vigilance, inhibition, and negativity/pessimism are evident in people with cluster C (Flink et al., 2019). This is supported by findings of more negative emotions connected with the behavioral inhibition system (BIS) (Gray, 1987; Meyer and Carver, 2000) such as fear and sadness (Caseras et al., 2001; Meyer, 2002; Lenzenweger and Willett, 2007; Karterud et al., 2016; Jarnecke et al., 2017; Frederiksen et al., 2021), also when they are alone (Gadassi et al., 2014). People with a diagnosis of cluster C are more prone to feeling shame (Kunst et al., 2020), shame being more painful, and more aversion to feeling shame even when controlling for lack of positive emotions and more negative emotions in general. Cluster C is especially associated with high levels of shame aversion, which leads to avoidance of potential situations where they anticipate feelings of shame. This avoidance seems to explain the correlation between being prone to feeling shame and cluster C (Schoenleber and Berenbaum, 2010; Currie et al., 2017).

People diagnosed with AvPD have more activation in the amygdala and report feeling more anxious when exposed to images of negative social interactions than others do. They show more amygdala activity and report more anticipation anxiety (Denny et al., 2015). This may show that they experience more anxiety in adverse social situations, and when anticipating negative social interactions. Which can explain excessive avoidance of social situations and withdrawal (Parker et al., 2000). Avoidance might be isolation, procrastination, self-harm, overeating, excessive computer use, or binge-watching TV – behavior that diverts attention from painful emotions, unmet needs, and potential failure (Coon, 1994; Gilbert and Gordon, 2013; Videler et al., 2017; Bachrach and Arntz, 2021). An active BIS creates a sensitivity to punishment that can cause avoidance. People diagnosed with AvPD are found to react strongly to punishment and loss, and quickly change course (avoid) when this happens (Caseras et al., 2001; Berenson et al., 2021). Reducing cost estimation predicts recovery (Borge et al., 2010), in concordance with an overactive BIS. Avoidance might solve a problem and reduce anxiety but also seems to elicit painful emotional states, as avoidant behavior is found to precede episodes of self-harm (Snir et al., 2015). These feelings typically inhibit behavior and foster withdrawal, which is an obstacle to experiencing agency. This characteristic way of experiencing primary emotions, accounts for 19% of variance in AvPD criteria, but less in other PDs except BPD (Karterud et al., 2016), suggesting that this is an important part of understanding AvPD.

There are few studies describing therapeutic work with discrete emotions. When it comes to inhibiting affects, therapists report emotional contagion – feeling shameful, insecure, and withdrawn in sessions (Pettersen et al., 2021). Therapy studies mainly describe work to increase anger, interest, and joy (activating affects). Colli et al. (2014) describe therapists also feeling both anger and optimism on behalf of their patients, even though the patients have little access to these emotions. This could be an important source for exploring the patients’ emotions. One case study reduces alexithymia and highlights the expression of anger and self-assertion in group therapy as a key experience (Inchausti et al., 2022). In a process study patients’ feelings of optimism after a session predicts a later decrease in sadness, and anger predicts more feelings of anger – maybe indicating increased self-assertiveness, but optimism also predicts subsequent less optimism (Hoffart, 2018). Maybe reflecting disappointment when allowing hope.

Little access and integration of the feeling interest could be a problem in therapeutic development, as it is an important emotion involved in learning and growth, closely linked to creativity, which may be an essential part of a therapeutic process (Frederiksen et al., 2021). Both interest and agency are about exploring the world. Lack of interest could make it more difficult to know what they value in the therapeutic process, and to influence the therapy. It could also make it more difficult for the therapists to understand what the patients want and how to help them act. In this way, little access to interest could be a barrier to being an agent in therapy and developing agency in daily life. Gordon-King et al. (2017), Bo et al. (2019), and Doomen (2018) emphasize that therapists should encourage engagement in joyous activities. New activities evoke emotions, which can be discussed in therapy for increasing awareness. Noticing positive emotions is inspiring, and as they discuss fantasies and dreams, leisure activity increases, and connecting the activities to emotions and wishes becomes easier (Gordon-King et al., 2017; Bo et al., 2019). In ST with drama (DT), the pretend mode of acting enables play and spontaneity. Schema modes of healthy adult (responsible) and free child (play) increase. DT also seems to provide a safe form of exploring feelings and expressions, as expressions of vulnerability and anger increase. Avoidant, submissive, and critical modes decrease (Doomen, 2018). Not too different from how children explore and develop their sense of self through play. More inhibiting affects correlate with a lower sense of self and increased activating emotions and decreased inhibiting emotions during therapy correlate with a stronger sense of self (Schanche et al., 2011; Berggraf et al., 2014b).

### 3.2. Disappearing in relationships

This theme is about how people diagnosed with AvPD typically interact with other people, and what happens with their agency when they are together with others. Relationships with important others are essential to the development of the self (Fonagy et al., 2002). We argue that relationships tend to weaken agency in people diagnosed with AvPD, but that they could be potential sources of developing agency – through the subthemes of fearful attachment and lack of self-assertiveness.

#### 3.2.1. Longing for connection, dreading to get close

People diagnosed with AvPD have significantly more attachment insecurity than what is found in general (Wilberg et al., 2023). Fearful attachment is characteristic of this disorder, with both high attachment anxiety and high attachment avoidance (Meyer et al., 2004; Eikenæs et al., 2016). The former is fear of abandonment and rejection from others, and excessive need for affirmation and distress when the person is unavailable. The latter is a reluctance to enter relationships with other people, or emotional distancing in relationships, keeping the relationship superficial. Attachment anxiety correlates with a tendency to perceive others as less friendly (Meyer et al., 2004) and problems in boundaries of self and others, which involves emotional contagion and difficulties in feeling separate from others (Beeney et al., 2015). Attachment avoidance and AvPD are directly linked (Beeney et al., 2015). They show a typical avoidant attachment pattern where they believe others to be less available for emotional support (Flink et al., 2019; Kunst et al., 2020), and show less reduction in dissociation and isolation after social contact (Gadassi et al., 2014), perhaps indicating not being able to use relationships for emotion regulation.

Attachment avoidance might be a coping mechanism for fear of abandonment if they become close to others (Hyman and Schneider, 2004). This may indicate an attachment conflict, where mixed affective reactions and expectations of negative emotional reactions hinder seeking social contact and fulfilling the natural needs of belonging and acceptance. This is supported by Gadassi et al.’s (2014) findings where social encounters are associated with more positive feelings and fewer experiences of isolation and rejection, but anxiety and shame increase significantly at the same time. They tend to want and long for social contact, but they feel doomed to be alone, as they dread others’ opinions. Longing for being alone but fearing loneliness at the same time (Hofmann, 2007; Sørensen et al., 2019a). There is a positive correlation between status, wealth, and AvPD in a population study, but a negative correlation with successful intimate relationships – perhaps suggesting that relational goals are harder to reach (Ullrich et al., 2007). Compared to social phobia, avoidance in AvPD seems to be more of closeness in relationships than of performing (Eikenæs et al., 2013).

People diagnosed with AvPD might seem cold and distant (Hummelen et al., 2007; Lenzenweger and Willett, 2007; Solomonov et al., 2020; McCloskey et al., 2021). This could be related to not expressing emotions, as alexithymia, and focusing on external events rather than internal states are correlated with more attachment avoidance. Difficulties in experiencing emotions are correlated with higher levels of attachment anxiety (Simonsen et al., 2020). This may indicate a link between attachment difficulties and alexithymia. Social dysfunction is also mediated by being too accommodating (Hummelen et al., 2007; McCloskey et al., 2021). Maybe they navigate their fear of dismissal in relationships by keeping a distance from strangers (attachment avoidance), and overly accommodating in close relationships (attachment anxiety). In this way, a fearful attachment pattern seems to be a barrier to being an agent, as they tend to want connections, but at the same time fear them and avoid them. Acting differently (cold) from what they need (to be close) and subordinating their own needs in relationships in order not to be abandoned. With that said, it is important to acknowledge heterogeneity of attachment styles within AvPD, where people also are found to have preoccupied, dismissing and secure attachment styles (Eikenæs et al., 2016).

Being mirrored by others, especially in attachment relationships, is an important way of getting to know yourself (Fonagy et al., 2002). Not being authentic in the presence of others can thwart the opportunity of mirroring, and create a sense of being alien or isolated (Coon, 1994; Hoffart, 2018; Flink et al., 2019), false, not separate, or not even present (Sørensen et al., 2019a). People diagnosed with AvPD often feel less recognized by others (Eikenæs et al., 2013). Those who can identify their emotions feel more recognized (Normann-Eide et al., 2015; Simonsen et al., 2020). It is important for the sense of self to feel recognized. At the same time, poor identity integration is the strongest predictor of difficulties in social functioning (Kvarstein et al., 2021), perhaps indicating a bidirectional relationship where the sense of self increases when recognized, but a poor sense of self makes it difficult to engage meaningfully in relationships, keeping agency low.

Therapy literature emphasizes the benefits of the relational context of therapy and to try to empower patients to interact in new ways in their daily life to become more secure. Therapists also perceive patients diagnosed with AvPD as emotionally distant (Colli et al., 2016). In ST the most frequent schema mode for people diagnosed with AvPD is the avoidant/detached coping mode, which appears 74% of the time in a therapy process study. This mode also fluctuates the most throughout therapy, which can indicate therapeutic changes (Peled et al., 2017). Therapy offers a social context to mirror and amplify experiences and emotions, to see themselves from an outside perspective, and get to know themselves better (Doomen, 2018). Self-disclosure predicts improvement in interpersonal therapy (Borge et al., 2010), and practicing emotional expression through bodily awareness also increases feelings of connectedness between therapist and patient (Simonsen et al., 2019). Patients diagnosed with cluster C with cold-submissive interpersonal problems improve more when the alliance is good (Ryum et al., 2010b). Meeting others in the same situation in group therapy helps participants feel community and provides support to try out new interpersonal behaviors (Alden, 1989; Renneberg et al., 1990; Sørensen et al., 2019b; Bachrach and Arntz, 2021). At the same time, group therapy elicits anxiety and dropout (Simonsen et al., 2022).

Two RCTs investigate the outcomes of ET and SST. They become less skeptical and perceive others as less hostile, report a significant change in withdrawal, have greater satisfaction with social situations, and have more self-confidence in handling targeted social situations (Alden, 1989; Stravynski et al., 1994). Fear of negative evaluation changes the most after group therapy with emotion regulation, positive feedback, and SST (Renneberg et al., 1990). Participants in an intimacy focus group have more social encounters and are more satisfied with them, but also have higher dropout (Alden, 1989).

#### 3.2.2. Letting others decide

People diagnosed with AvPD mainly have interpersonal difficulties concerning lack of agency: Lack of self-assertion (Hyman and Schneider, 2004; Leising et al., 2006; Hummelen et al., 2007; Frandsen et al., 2020; McCloskey et al., 2021), being too accommodating, easily exploited (Hummelen et al., 2007), pleasing others (Nordahl and Stiles, 2000), and being socially withdrawn (Solomonov et al., 2020; McCloskey et al., 2021). They are described with difficulties asking for help (Inchausti et al., 2022), a tendency to subordinate their needs to others (Hyman and Schneider, 2004; Dimaggio et al., 2017; Flink et al., 2019), and impaired limits (Flink et al., 2019), making them dependent on other people’s happiness and keeping their own needs out of sight. They describe that they can have clear opinions when they are on their own, but that their opinions disappear in the face of others. Except when being with a trusted other for example in nature – then they can make decisions such as where to put up a tent. They do what other people say and feel that others take advantage of them, which ignites unexpressed anger (Sørensen et al., 2019a). It is more difficult for them to have a stable sense of self when they are together with other people (Simonsen et al., 2020).

Submissiveness also occurs in therapy (Colli et al., 2016). They report entering therapy with expectations of getting an explanation, guidance, and relief. They wait to see whether the therapy helps. After a while of being told what to do, the therapy starts to feel like being handled, and they feel misunderstood, passive, and discontent. They can have difficulties understanding the therapists’ techniques, but they dare not question them and pretend that the therapy works (Sørensen et al., 2019b). It can be difficult to notice ruptures as patients give little or disguised signals, such as not doing the work or missing the appointments, avoiding confrontation, and submitting to the therapists’ wishes. Missing a rupture can make patients feel unrecognized and worsen the therapeutic relationship (Cummings et al., 2011).

How should therapists meet the patients’ submissiveness when it appears in the sessions? This is important to address as growth in the sense of self seems to predict less cold, avoidant, and submissive interpersonal problems (Berggraf et al., 2014a), and client factors such as engagement and bringing up important themes characterize successful therapies. While unsuccessful therapies are characterized by therapist variables such as being directive and taking control (Lilliengren et al., 2019). Therapists tend to react with rigidity to ruptures and attribute the rupture to the patients’ pathology – e.g., negative schema of the therapists, which can feel invalidating for the patients (Cummings et al., 2011). Ryum et al. (2010a, 2022) find the alliance correlates with better interpersonal functioning, but the therapists’ competence in assigning homework has a larger correlation. These variables are unrelated. The therapist setting an agenda correlates with homework, but also with negative outcomes. Perhaps setting an agenda is positive when it means that therapists follow up on the everyday changes patients are making, but not if therapists become too rigid and control the sessions without regard for the patients’ agency in sessions. Interpretations (defined as suggestions of the unconscious meaning behind patients’ experiences) are also negatively associated with patient-therapist affiliation and outcome (Schut et al., 2005). Which can support the hypothesis that a too directive therapist style might be unfortunate with this group. Addressing submissiveness in sessions seems to be a difficult task. When the alliance is poor, transference interpretations (interventions addressing feelings and interplay between the patients and therapists) correlate with poorer interpersonal functioning as an outcome. A moderate degree of transference interpretations where alliance is good however, correlates with better interpersonal functioning as an outcome (Ryum et al., 2010b). At the same time, Høglend et al. (2011) found a significant decrease in Cluster C symptoms with transference interpretation compared to therapy without this intervention. To summarize, reflection upon interpersonal events between therapists and patients seems to facilitate agency, but only to a moderate degree in the context of a good alliance.

Josephs et al. (2014) describe a successful confrontation of relational difficulties in the therapeutic relationship in their case study: When the therapist confronts the patient with her help-rejecting complaining stance and her passivity’s role in her interpersonal problems, helps her find new ways of solving them and supports her engagement, the patient uses more adaptive defenses such as self-observation. She can self-reflect on how her help-rejecting complaining serves her poorly. Perhaps showing how identifying maladaptive interpersonal patterns, working on new social skills, and receiving support can increase the sense of agency – from a passive position (help-rejecting complainer) to becoming an agent (self-reflection and attuning behavior to reach goals). This is also supported by another case study, showing a significant decrease in disavowal as a defense (Presniak et al., 2010). An increase in self-reflection, again increases the therapist’s confrontations of the patient’s lack of agency (Josephs et al., 2014). This demonstrates how the patient’s level of agency also affects the interaction in therapy.

How to repair a poor alliance if talking about it can make it worse? Patients with cluster C have greater improvement in psychotherapy when therapists get feedback on outcomes weekly than without feedback (de Jong et al., 2018). Perhaps demonstrating the help they potentially get from feedback measures in letting therapists know how they are doing. Patients who experience ruptures followed by repair in the therapeutic alliance have better personality development than in those cases where there are no ruptures to repair. Episodes of repair are less likely with more severe PDs and interpersonal problems (Strauss et al., 2006). People diagnosed with AvPD describe developing agency when therapists meet them with curiosity (Sørensen et al., 2019b; Pettersen et al., 2021). This therapeutic stance seems more productive for supporting them to speak up and influence the therapists, compared to making inferences about conflict in the therapeutic relationship. This is supported by the fact that therapist flexibility in the face of difficult interactions at the start of therapy is significant in successful therapies (Lilliengren et al., 2019). Perhaps this gives a message to patients that they have an impact on the relationship – giving a sense of agency. Though therapists describe a curious investigation of the patient’s state of mind to elicit annoyance and self-doubt (Pettersen et al., 2021). Therapists report that when they give the patients responsibility for directing the sessions, this can be too difficult and create a withdrawal. Some structure and instructions seem necessary for the patients to be able to come forward in the sessions (Pettersen et al., 2021). Development in therapy can create fear and a sense of having to manage on their own, which can make the relationship with the therapist seem distant again (Sørensen et al., 2019b).

Group therapy can also give important corrective emotional experiences with being assertive and developing agency (Renneberg et al., 1990; Feske et al., 1996; Bachrach and Arntz, 2021). Gude and Hoffart’s (2008) and Hardy et al.’s (1995) findings suggest CBT is superior in helping patients diagnosed with cluster C with non-assertiveness (and self-esteem in Hardy et al.’s case) (among other interpersonal problems) compared to DT. Perhaps this is because of the emphasis CBT has on making changes in everyday life, not just in therapy. Patients living alone make less improvement than patients in a relationship (Kvarstein et al., 2021), and patients with little interpersonal distress are less open and engaged in therapy and have fewer realizations (Kolden et al., 2005). Positive changes in social functioning and personality functioning are reported after combined group MBT and individual MIT, with larger changes in the self-domain than the interpersonal domain (Wilberg et al., 2023). Perhaps reflecting agency as an important starting point for change before an interpersonal change.

### 3.3. A weak sense of self

This theme describes people diagnosed with AvPD’s sense of self, which we define as contact with inner experience, a self-accepting attitude, and the belief that you can pursue and achieve your goals and needs (Ulvenes et al., 2014). Sense of self encompasses being a part of a social context, and having a self-narrative that gives meaning to life changes through a coherent story of life events and responses in the past, present, and future that gives a stable sense of being the same person (McAdams and Pals, 2006). Sense of self develops through exploring and accepting emotions, being accepted by others, and experiencing mastery (Fonagy et al., 2002). The ability to read your mind and be able to act confidently accordingly is at the core of the definition of agency (Rennie, 2000, 2001). In this way, we view the sense of self as including agency and as the output of the two preceding themes. We argue here that people diagnosed with AvPD have fundamental difficulties in this area, through the subthemes of poor narratives, and a doubtful and relentless self.

#### 3.3.1. Diffuse stories of who they are

People diagnosed with AvPD have been found to have unstable self-images (Eikenæs et al., 2013). Both case studies and larger samples find low self-reflection, self-respect, self-knowledge, meaning in life, and identity integration (Eikenæs et al., 2013; Simonsen et al., 2019; Kvarstein et al., 2021; Valikhani et al., 2022). Generating stories of who we are binds the past to the present and future imagined selves. This is an important ability to create stability of the sense of self through time and across situations (McAdams, 2013). Stories about the self often entail information about important life goals of agency and communion (motives of affiliation and social belonging) (Mansfield and McAdams, 1996). Difficulties with creating such narrative identities are a part of the picture in many psychopathologies, including PDs (Dimaggio et al., 2009; Lind et al., 2020; McAdams, 2020; Lind, 2021). An important ability to be able to build a narrative identity is autobiographical memories and autobiographical reasoning, as episodes in life are the building blocks of the storied selves, and autobiographical reasoning is the process of tying the episodes together and connecting them to each other and the self in a meaningful and coherent manner (Dimaggio et al., 2009; Lind et al., 2020; McAdams, 2020; Lind, 2021). Memory difficulties could explain some of the lack of self-coherence found in AvPD. They have fewer memories in general and specifically less autobiographical than semantic memories. Semantic memories are generalized facts such as “I am a boring person,” and autobiographical memories are specific episodes with senses, feelings, thoughts, and dialogue like “one time, I felt like I was boring my friends talking about an interest they did not share with me.” The tendency to ruminate and worry in a self-centered and analytic way facilitates overgeneralized thinking and thwarts the mindful presence needed to form autobiographical memories (Spinhoven et al., 2009). This may lead to overly negative and generalized “truths” about who they are, and little sense of continuity.

The deficit in creating a coherent story about their past to inform their current sense of self is accompanied by difficulties in having a clear sense of their future selves - lacking connection with their motives and needs (Chan et al., 2015; Simonsen et al., 2019; Bachrach and Arntz, 2021). They see themselves as less able to reach their goals (Meyer and Carver, 2000; Meyer, 2002; Flink et al., 2019), as they evaluate themselves as less self-controlled, competent, dutiful, achievement-striving, and self-disciplined (Hummelen et al., 2007; Flink et al., 2019). Although compared to people without AvPD, they do not lack drive – the persistence in pursuit of incentives (Meyer, 2002). People diagnosed with PDs are in general found to have more negative stories about their lives, where they portray themselves as less agentic, more a victim of their circumstances, with thwarted wishes for communion and with less coherent and specific narratives (Lind et al., 2020). Although most of this research was done with BPD, similar findings were presented in a case study of narrative identity with AvPD (Lind et al., 2021). Rettew et al. (2003)‘s findings of people with AvPD retrospectively reporting less athletic achievement, hobbies, popularity, and fewer positive relationships with adults in adolescence is in accordance with this. Being out of touch with their wishes, unable to pursue needs, and acting contrary to their needs damage their sense of agency as it builds on perceiving their actions as a product of their intentions (Rennie, 2000, 2001). Difficulties in having a coherent sense of self and setting and attaining goals could be due to alexithymia, as this correlates with difficulties in self-reflection and emotions are important for consolidating memories and setting goals (Sørensen et al., 2019a; Simonsen et al., 2020). Attachment anxiety, low self-confidence, difficulties with self-assertion and maintaining boundaries can also explain an unclear sense of self and future – as it makes emotional clarity and clarity in their opinions particularly difficult when together with others (Beeney et al., 2015; Sørensen et al., 2019a; Inchausti et al., 2022).

Learning to create a personal narrative is an important task in therapy, as it is a part of learning to self-reflect and develop a mature identity (McAdams and Pals, 2006; McAdams, 2013). Only scratching the surface of their problems without a deeper exploration of feelings and the relational origins could create frustration in the patients (Cummings et al., 2011). Although a case study describes that even when asked directly by their therapists, patients may have considerable difficulty with self-reflection (Dimaggio et al., 2007). At the same time, making a narrative together in therapy can help the patients understand their past actions in relevant contexts, and enable them to feel free in doing things differently now (Hyman and Schneider, 2004; Videler et al., 2017, 2018; Sørensen et al., 2019b; Lind et al., 2021). This also applies to the therapeutic relationship. Alliance ruptures can be dealt with by referring to similar important relational events in the past, and trying to do something different together, such as in a role play (Cummings et al., 2011). Problems with being an agent can create confusion in therapy (Simonsen et al., 2019), as the therapeutic interaction needs to be guided by the patient’s treatment goals. It is difficult to answer questions about goals for therapy – what is the right answer (Sørensen et al., 2019b)? Some research has documented wishes people diagnosed with AvPD can have for therapy. They want to find the courage to resolve fear and uncertainty, get to know who they are with likes and dislikes, feel happy, and be able to provide for themselves without fearing others’ judgment. They want to be able to be steady when facing adversities (Sørensen et al., 2019b), express themselves (Hyman and Schneider, 2004) and live a normal life with a job, a home, and relationships – being independent (Hofmann, 2007).

Guide for narrative repair (GNaR) is a tool for therapists to help patients detect the above mentions difficulties with narrative identity and develop a more positive and supportive narrative that can unite difficulties with hope and strengths to empower patients to envision a more hopeful future. This is perhaps one of the most explicit ways of helping patients with more coherent and positive life stories and could be combined with other therapeutic methods (Lind et al., 2021). Other methods also describe how to search for strengths and create coherency: ACT combined with DBT, ST, and MIT explicitly frame avoidance as hindering people from living according to their values (Chan et al., 2015) or needs (Dimaggio et al., 2017; Gordon-King et al., 2019; Bachrach and Arntz, 2021). They propose helping patients to assert their motives, and to change cognition and behavior that stops them from attaining their goals. A patient in a case study experienced that enduring feeling foolish to reach her goals made her feel more efficient and self-confident (Chan et al., 2015). Studies describe increased self-value, mastery, flow, and development when exploring interests and activities such as computer support (Videler et al., 2017), nature, animals, playing music, or doing art (Dimaggio et al., 2017), but they evaporate by the thought of others evaluating their actions (Sørensen et al., 2019a). In group therapy for SST, the participants reported it to be important to decide on social situations for training (Alden, 1989; Stravynski et al., 1994). After the intervention, the participants were more able to pursue their social wishes (Stravynski et al., 1994). Stålsett et al. (2010, 2012) find that group therapy focusing on affect consciousness, existential issues, relationship to religion, and making a narrative with cluster C patients increases self-representation, through less submissiveness and over-conscientiousness. Perhaps indicating more meaning in life and a greater capability to live by their values.

Simonsen et al. (2019) describe emerging confidence in a case study when uniting conflicting self-views of a critical person in control or an emotionally vulnerable person – accepting multi-faceted parts of the self. Case studies describe two common opposing sides in people diagnosed with AvPD – one self-critical striving for autonomy and one vulnerable and sad containing the need to belong. They describe how experiences of understanding and validation of vulnerability in daily life and group therapy – gradually develop self-assertiveness (Bachrach and Arntz, 2021; Inchausti et al., 2022). Resolving the dichotomy between belonging and autonomy. Perhaps AvPD contains a conflict between these two needs when perceiving others as critical – but when others accept their vulnerability, mastery and the need for others seem more compatible.

#### 3.3.2. Self-doubt

Studies describe people diagnosed with AvPD as having an inhibiting doubtful way of being. Ruminating (van Rijsbergen et al., 2015), questioning the validity of their own experiences (Sørensen et al., 2019a; Inchausti et al., 2022) and only briefly, if at all, certain in decisions (Coon, 1994; Simonsen et al., 2019). They value certainty and experience new situations as frightening and overwhelming (Alvarez-Ramirez, 2015). They think everyone else is more certain than them, and struggling to do even daily tasks, such as exercising, if it is not given how they are supposed to do it (Sørensen et al., 2019a). Overthinking could be a way of avoiding active problem-solving, as they often feel unable to cope with stressors (Jarnecke et al., 2017), and handle difficulties with less solution-focused strategies (also characteristic for other PDs) (Arntz et al., 2011). Self-efficacy and community activism are low, and they often feel powerless (Olssøn and Dahl, 2012). Alexithymia correlates with an unstable sense of self and could explain some of the doubtfulness and vagueness in their self-experience (Simonsen et al., 2020). Self-esteem seems normal in the face of supportive comments from others, but in the face of subtle critique, self-esteem is significantly lower than in healthy controls (Hyman and Schneider, 2004; Bowles et al., 2013). This can show the contingency of their self-esteem on others’ opinions of them, making their sense of self blurrier in social situations. This could be one of the causes behind the extensive social avoidance in this disorder - withdrawing to preserve some self-coherence. Paradoxically, the lack of social interaction could make it more difficult to know who they are and what they want.

Therapists perceive that patients doubt their ability to change certain parts of themselves, even if they try (Pettersen et al., 2021). Increasing emotional awareness (Simonsen et al., 2019), mastering social situations (Boettcher et al., 2019; Gordon-King et al., 2019), and practicing decisions about therapy (Coon, 1994) are suggested interventions to reduce self-doubt. Changes that do not come from within do not give any sense of mastery, and can make them feel more distant and less able to influence the therapy (Sørensen et al., 2019b).

#### 3.3.3. Self-critique

Perhaps contributing to the above-mentioned uncertainty is a tendency toward harsh and negative self-talk. Dysfunctional beliefs held with certainty are common in people with PDs (Dreessen et al., 1999; van Rijsbergen et al., 2015). For people diagnosed with AvPD, this is often negative self-cognitions such as being a failure and lack of self-control (Hyman and Schneider, 2004; Gilbert and Gordon, 2013; Videler et al., 2017; Flink et al., 2019; Kunst et al., 2020). When experiencing painful emotions, they react with negative thinking (van Rijsbergen et al., 2015) and negative schema beliefs can induce negative feelings such as anger and sadness (Hoffart, 2018). When problem-solving, their cognitions are characterized by negative emotions and self-critique (Arntz et al., 2011). Excessive rumination often leads to a conclusion where they find that something is wrong with them (Sørensen et al., 2019a). These negative cognitions seem to deteriorate agency and foster underachievement (Coon, 1994); “I will not be able to do things my way, and I will fail, why bother trying” (Videler et al., 2017). They hold back in fear of others’ critique (Nordahl and Stiles, 2000; Arntz et al., 2011; Bowles et al., 2013; Eikenæs et al., 2013; Rees and Pritchard, 2015; Berenson et al., 2018; Flink et al., 2019). Self-critique rapidly follows attempts to articulate needs, keeping agency low (Inchausti et al., 2022). The perception of others as critical can be due to difficulty with decentralizing – reading others’ minds as unrelated to their mind (Moroni et al., 2016). They interpret others as critical because of their self-criticism.

Negative beliefs correlate with low self-esteem (Dreessen et al., 1999). They are found to have low self-esteem (Meyer, 2002; Lynum et al., 2008), little self-respect and self-confidence (Eikenæs et al., 2013), and a negative self-image (Feske et al., 1996; Hofmann, 2007). Negative thoughts about the self have paralyzing and deteriorating effects on even basic self-care such as nourishment and hygiene (Videler et al., 2017; Bachrach and Arntz, 2021). Self-devaluation frequently preceded episodes of self-harm (Snir et al., 2015). One study found a connection between AvPD and less self-compassion (Valikhani et al., 2022). Some studies depict a discrepancy between strong self-critique and impossible ideal selves or unrealistic standards, which may illustrate that perfectionism is a way of coping with feeling like a failure (Coon, 1994; Hofmann, 2007; Dimaggio et al., 2017; Flink et al., 2019; Gordon-King et al., 2019; Sørensen et al., 2019a). It could also be part of a more complex diagnostic picture of vulnerable narcissism with underlying great expectations of themselves and others to compensate for low self-esteem and withdrawing in fear of disappointment (Dickinson, 2002; Dickinson and Pincus, 2003).

CBT (including ST) is probably the most explicit in addressing negative cognitions. Different studies of ST find a decrease in maladaptive schemas and an increase in adaptive schemas (e.g., Videler et al., 2017, 2018; Peeters et al., 2021), a decrease in schema beliefs, and more optimism (Hoffart, 2018). Reducing negative self-cognitions can significantly increase self-confidence (Hyman and Schneider, 2004; Gilbert and Gordon, 2013). Negative thoughts are described as still present, but more balanced after therapy (Gordon-King et al., 2019). However, negative self-image changes significantly less over the course of therapy with AvPD compared to social phobia (Feske et al., 1996). Videler et al. (2017) describe waiting to work with negative schemas until the alliance is strong and start working with accessing and strengthening positive schemas by focusing on good experiences from work or relationships. In a case study of CBT, they had to work on the core assumption of being inferior for exposure to avoided situations and new social learning to take place (Rees and Pritchard, 2015). Renneberg et al. (1990) suggest working with negative self-image by getting positive feedback in a group and finding positive aspects of self. Therapists try to boost self-confidence through mastery without therapist-involvement (Bachrach and Arntz, 2021) and participants had better self-confidence after the social skills training group (Alden, 1989).

All these interventions prioritize new positive experiences with agency in a group and in their lives to reduce self-critique. This might be connected to therapists experiencing challenging negative thoughts as «hammering» the most vulnerable parts of their patients (Pettersen et al., 2021). Self-esteem is situational, and it could be pathological to have unchanging self-esteem regardless of whether it is high or low (Baumeister et al., 2003). Fluctuations in self-esteem actually seem to predict positive change in therapy (Cummings et al., 2012). Practicing emotional awareness and connection and experiencing social acceptance and mastery is an important route to strengthening the sense of self in general, rather than boosting self-esteem (Baumeister et al., 2003).

## 4. Discussion

Agency seems to be particularly low in people diagnosed with AvPD. Poor access to inner experiences, few activating emotions, many inhibiting emotions, and a low tolerance for emotions seem to be important psychological mechanisms that make it more difficult to know what they want and inhibit them in pursuing wishes (Schanche et al., 2011; Ulvenes et al., 2014; Berggraf et al., 2014b; Simonsen et al., 2020). Relationships seem to hinder rather than foster a sense of agency for people diagnosed with AvPD because of their fearful attachment where they long for connection yet dread to get close, but fear being abandoned – often resolved with withdrawal (Beeney et al., 2015; Eikenæs et al., 2016). The need for intimacy seems particularly conflicted and difficult to pursue, in addition to the tendency to subordinate to others (Frandsen et al., 2020; McCloskey et al., 2021) which can be seen as a way of coping with attachment anxieties. This leaves them with unclear signals to act upon from the inside and disturbing noise and little support from social surroundings that gives them an unclear sense of self with little cohesion, full of doubt and critique (Eikenæs et al., 2013; Kvarstein et al., 2021; Valikhani et al., 2022). This is a poor foundation for reflective and intentional actions.

Cyclic psychodynamics can help us understand how an avoidant manner of being becomes self-sustaining. The first attachment relationships create the first vicious cycle of lack of emotional validation and encouragement of curious exploration – creating reactions of withdrawal, not expressing themselves, subordinating to others, and undermining their emotions (Normann-Eide, 2020). This creates an uncertainty of the self and an unclear and withdrawn way of relating (Ulvenes et al., 2014). The fearful attachment pattern creates more vicious cycles in new relationships, where they come off as uninterested, cold, or pretend to be who they think others wish them to be (McCloskey et al., 2021). This hinders mirroring and real connection, leading to feelings of alienation and failure (Flink et al., 2019; Kunst et al., 2020). Meyer and Carver (2000) find supportive evidence for this pattern in correlations between recalling an aversive childhood, emotional sensitivity, and withdrawal in AvPD.

How can therapists help people diagnosed with AvPD break this vicious self-sustaining cycle? The research implies that they should help patients diagnosed with AvPD express their emotions, thoughts, and needs in an accepting atmosphere (Schanche et al., 2011; Ulvenes et al., 2014; Berggraf et al., 2014b). They should encourage the patients to do so also in other possible relationships in daily life (Ryum et al., 2010a, 2022) – to create virtuous cycles of emotional clarity, connectedness, and agency. Alliance ruptures could then be positive learning opportunities to turn the vicious cycles (Cummings et al., 2011). Group therapy could be an important arena for corrective emotional experiences (Alden, 1989; Renneberg et al., 1990; Sørensen et al., 2019b; Bachrach and Arntz, 2021), but at the risk of dropout (Simonsen et al., 2022). Considering the great role of avoidance and withdrawal in AvPD, perhaps the most important arena for change is the behavioral changes that patients do in their daily life settings in between sessions. It is important that therapists encourage and support these changes in the working alliance, such as by following up on homework and behavioral tasks (Dimaggio et al., 2020; Centonze et al., 2021; Ryum et al., 2022). Supporting clients diagnosed with AvPD to not just develop a positive relationship to the therapist, but experience change in their own actions can support agency greatly, as having goals, perceiving them as achievable, and experiencing mastery is key to developing sense of agency (McAdams, 2013). That patients take action toward recovery, and that therapists support this is an important factor in the effect of psychotherapy (Wampold and Imel, 2015).

Previous research has focused on developing and trying out different treatment models. Cyclical psychodynamics creates a broad framework to unite the different approaches emphasizing interventions in different parts of the cycle. DT emphasizes understanding the origin of the vicious cycle and breaking them through increased insight and new experiences in the therapeutic relationship. CBT (including ACT and DBT and SCT) emphasizes creating new virtuous cycles through becoming aware of the vicious cycles and new experiences in daily life. ST, MBT, and MIT intervene by understanding vicious cycles from childhood and encouraging patients to create new virtuous cycles both in therapy and in daily life. The goal of this article is not to evaluate these methods against each other, but to unite the contributions to create a deeper understanding of how therapy can support agency in people diagnosed with AvPD.

Cyclical psychodynamics postulate that the patients’ autonomy grows through the influence of the therapist (Wachtel, 2014). In the case of AvPD, how not to influence too much on someone too easy to influence? It would be interesting to investigate the therapeutic outcome of feedback tools in therapy, as these may ease the task for people diagnosed with AvPD of speaking up and influencing collaboration in therapy (de Jong et al., 2018). Facilitating emotional corrective experiences in the therapy – repeating destructive relational cycles with the therapist and experiencing new ways of interaction that can inspire the patients to influence their significant relationships in new ways. Which then can be supported by following up on behavioral changes outside sessions (Dimaggio et al., 2020). Following up on behavioral changes in daily life can also be a corrective relational experience of being supported in exploration and activity.

On that note, it would be interesting for future research to evaluate therapist qualities and outcomes with this patient group. Sørensen et al. (2019b); Pettersen et al. (2021) describe playful, humble, and self-revealing therapists, perhaps resembling therapist flexibility; one of the most important therapist factors from process-research in general (Owen and Hilsenroth, 2014). Lilliengren et al.’s (2019) findings of greater therapist flexibility at the beginning of successful therapies support this. Perhaps the use of feedback tools also could help therapists be flexible and let the specific patient influence their unique therapeutic process.

Self-related functions improve more than interpersonal functions (Simonsen et al., 2022; Wilberg et al., 2023) – it could be that this is evidence of the fundamental difficulties of agency in this disorder and that improving agency is an important starting point in therapy. This is also supported by Lilliengren et al.’s (2019) finding that patients were more engaged in successful therapies. Summary of research in mindreading abilities also suggest that self-reflection is important to be able to understand the thoughts and emotions of others in general (Dimaggio et al., 2008). Perhaps even more interesting than therapist qualities would be to research patient qualities and outcomes with this patient group, especially regarding agency. To our knowledge, previous research has not investigated this. More research into what people diagnosed with AvPD do to get better in their daily life would also be in line with the importance of homework and behavioral change (Dimaggio et al., 2020; Centonze et al., 2021; Ryum et al., 2022).

The power of the alliance in creating successful outcomes of therapy lies in the therapeutic relationship enabling the patient to confront their fears and elicit responses in others in their daily life that make them feel good about themselves – strengthening their agency in their everyday life (Wachtel, 2014). We therefore highlight that future research should look into therapeutic work (both patients, therapists, and the relationships) and how it affects patients’ agency in daily life. Wachtel (2014) argues that to understand how therapists can strengthen patients’ sense of agency, we need to understand more of the details of the communication in the relationship – who says what, how is it said and what impact does it have on the other person? Qualitative and process research would be well suited for this. Talia et al. (2014) give one example of this type of research, where they analyze patients’ in-session discourse for proximity regulating behavior in relation to their attachment styles. A difficulty in studying the nature of the therapeutic relationship with AvPD is whether we would get authentic reports, as people diagnosed with AvPD are found to give high alliance scores (Simonsen et al., 2022), but this could be an inauthentic pseudo alliance to avoid conflict (Sørensen et al., 2019b).

### 4.1. Future understanding within ICD-11 and DSM-5 AMPD diagnostic frameworks

Trying to understand what it is like to struggle with AvPD through the lens of psychological mechanisms and relational dynamics that facilitate, or hinder agency could help us continue to identify, understand and treat these difficulties also in the new diagnostical framework of ICD-11 (World Health Organization, 2023) and AMPD (American Psychiatric Association, 2013). The overarching theme of the article about how agency might be thwarted in AvPD corresponds to the self-function-criteria of self-direction. The theme of “diffuse stories of who they are” corresponds to the difficulties these patients might experience with the criteria of stability and coherence of sense of identity. The themes of “self-doubt,” “self-critique” and “lacking access to inner life” might correspond to the self-function criteria of maintaining an overall positive and stable self-worth and having accurate perception of own characteristics, weaknesses, and strengths. The theme “longing for connection, dreading to get close” corresponds to the interpersonal functioning criteria of interest in engaging in relationship with others. The criteria of ability to understand others’ perspective can be understood in the light of the theme “self-criticism” and how easily the interpretation of others is colored by this perspective. The main theme of “disappearing in relationships” can help us understand what difficulties people with AvPD might experience in the criteria of developing and maintaining mutually satisfying relationships. The theme “letting others decide” helps us to identify a pattern in the criteria of handling conflict. To evaluate emotional manifestations of the disorder we can look to the main theme of “painful and joyless feelings without a name.” To evaluate appraisal patterns under stress we can look to the themes of “self-doubt,” “self-criticism” and the main theme of “disappearing in relationships.” The behavioral reaction of withdrawal described as connected to all the main themes of this article corresponds to the criteria of evaluating behavioral pervasiveness of the disorder. The trait domains of negative affectivity and detachment captures much of the described difficulties with emotions, negative self-view and relational distance described in this paper (Bach and Eikenæs, 2021; Bach et al., 2022). In the same way, the corresponding themes of this article can help us identify ways of facilitating recovery from AvPD with the described therapeutic issues and suggestions in the end of each theme, such as working on narrative identity is a way of addressing coherence of self (Lind, 2021).

### 4.2. Limitations

This narrative review seeks new lines of investigation based on previous findings. The readers should not read the article claiming the current state of knowledge in the way that a systematic review does but as the authors’ suggestions of possible understandings to continue exploring. There are also several limitations to the generalization of the findings presented in this article. First and foremost, people diagnosed with AvPD are as different as people are in general, and it is a condition that entails considerable heterogeneity. In our attempt to describe trends in ways people might experience their agency, this heterogeneity is not represented. A substantial part of the literature is case studies. Many of the studies have small sample sizes. Most of the studies have primarily female samples, which may weaken the results’ applicability to men. Some of the correlational studies study avoidant personality traits in normal samples. While many of the therapy studies have high comorbidities, complicating whether difficulties are specific to AvPD, or they control for many comorbid variables, making the studies less ecologically valid. Both Bamelis et al. (2014) and de Jong et al. (2018) use subgroup analysis, which entails high uncertainty and the need for further study. Many of the studies of affect compare AvPD with BPD (e.g., Johansen et al., 2013; Frederiksen et al., 2021). Significant differences in experiencing different emotions could be due to people with BPD experiencing particularly much of this feeling and should therefore be interpreted with this in mind. We argue that the article has transferability to the degree that it can be relatable for people who have, work with, or relate to people diagnosed with AvPD.

## 5. Conclusion

Poor agency appears as one of the elements of core pathology of AvPD and consequently it needs to be considered a treatment target. Connecting with activating emotions and creating experiences with mastery, influence, and acceptance in social contexts of therapy and daily life may be key to strengthening agency in this group.

## Author contributions

AW searched the literature and did the initial analysis with quality assurance from P-EB. AW wrote the first draft of the manuscript. All authors contributed to the conception and design of the study, subsequent rounds of analysis, the manuscript revision, and read and approved the submitted version.

## Funding

The Research Council of Norway provided financial support for this project (grant number 336858). Voss DPS NKS Bjørkeli AS paid the publication fee to support open-access publishing.

## Conflict of interest

The authors declare that the research was conducted in the absence of any commercial or financial relationships that could be construed as a potential conflict of interest.

## Publisher’s note

All claims expressed in this article are solely those of the authors and do not necessarily represent those of their affiliated organizations, or those of the publisher, the editors and the reviewers. Any product that may be evaluated in this article, or claim that may be made by its manufacturer, is not guaranteed or endorsed by the publisher.
